# Sialic acid plays a pivotal role in licensing *Citrobacter rodentium’s* transition from the intestinal lumen to a mucosal adherent niche

**DOI:** 10.1073/pnas.2301115120

**Published:** 2023-07-03

**Authors:** Qiaochu Liang, Caixia Ma, Shauna M. Crowley, Joannie M. Allaire, Xiao Han, Raymond W. W. Chong, Nicolle H. Packer, Hong Bing Yu, Bruce A. Vallance

**Affiliations:** ^a^Division of Gastroenterology, Hepatology and Nutrition, Department of Pediatrics, BC Children’s Hospital Research Institute and the University of British Columbia, Vancouver, BC V5Z 4H4, Canada; ^b^ARC Centre of Excellence for Synthetic Biology, School of Natural Sciences, Faculty of Science and Engineering, Macquarie University, North Ryde, Sydney, NSW 2109, Australia

**Keywords:** bacterial pathogens, intestinal mucus, sialic acid

## Abstract

Enteric bacterial pathogens pose significant threats to human health; however, the mechanisms by which they infect the mammalian gut in the face of daunting host defenses and an established microbiota remain poorly defined. For the attaching and effacing (A/E) bacterial family member and murine pathogen *Citrobacter rodentium*, its virulence strategy likely involves metabolic adaptation to the host’s intestinal luminal environment, as a necessary precursor to reach and infect the mucosal surface. Suspecting this adaptation involved the intestinal mucus layer, we found that *C. rodentium* was able to catabolize sialic acid, a monosaccharide derived from mucins, and utilize it as its sole carbon source for growth. Moreover, *C. rodentium* also sensed and displayed chemotactic activity toward sialic acid. These activities were abolished when the *nanT* gene, encoding a sialic acid transporter, was deleted (Δ*nanT*). Correspondingly, the Δ*nanT C. rodentium* strain was significantly impaired in its ability to colonize the murine intestine. Intriguingly, sialic acid was also found to induce the secretion of two autotransporter proteins, Pic and EspC, which possess mucinolytic and host-adherent properties. As a result, sialic acid enhanced the ability of *C. rodentium* to degrade intestinal mucus (through Pic), as well as to adhere to intestinal epithelial cells (through EspC). We thus demonstrate that sialic acid, a monosaccharide constituent of the intestinal mucus layer, functions as an important nutrient and a key signal for an A/E bacterial pathogen to escape the colonic lumen and directly infect its host’s intestinal mucosa.

## Significance

Intestinal mucus forms an important protective barrier, preventing nonpathogenic bacteria from escaping the gut and damaging the cells that line it. We show that bacterial pathogens have evolved strategies to overcome this barrier by first migrating toward sialic acid, a sugar derived from mucus. Upon acquiring sialic acid, pathogens use it to fuel their growth and expansion in the gut. Sialic acid also induces the secretion of two key proteins that help these pathogens penetrate the mucus, as well as stick to the cells lining the gut. Correspondingly, a pathogen unable to sense or utilize sialic acid is significantly impaired in infecting its hosts or causing disease. Thus, the metabolism of sialic acid plays a key role in bacterial pathogenesis.

The mammalian gastrointestinal (GI) tract is a complex environment, with the large intestine harboring huge numbers of commensal microbes that are largely confined to the gut lumen by physiochemical barriers, such as the epithelium and the overlying mucus layer. Over the course of the host’s development, the resident bacteria and other microbes colonize unique biogeographical niches within the colon, reflecting their abilities to not only withstand local environmental conditions but also obtain nutrients from their hosts or nearby microbes (1, 2). This complicated web of microbe–host interactions typically proves beneficial to both the microbes and their hosts (3–5). In contrast, since the gut is the easiest route for bacteria to enter their host (i.e. through ingested food or water), it is also the primary target for many enteric bacterial pathogens (6). To successfully infect their hosts, invading pathogens must not only subvert host defenses but also overcome colonization resistance by competing with the resident microbiota for limited nutrients and space (6, 7).

In the last decade, many advances made in the field of intestinal pathogen metabolism and colonization resistance have come from the study of *Citrobacter rodentium*, a murine bacterial pathogen related to the clinically important attaching and effacing (A/E) pathogens enteropathogenic *Escherichia coli* (EPEC) and enterohemorrhagic *E. coli* (8). A/E pathogens are best known for their intimate attachment to intestinal epithelial cells (IEC) via their type III secretion system (T3SS) (9, 10). Once adherent to the intestinal epithelial surface, *C. rodentium* can leverage IEC-derived H_2_O_2_ to drive its respiration and establish its own niche (11). However, prior to this phase of infection, ingested A/E pathogens reside within the gut lumen (8). We suspect that for A/E pathogens to successfully infect their hosts, they must adapt their metabolism to the luminal environment, acquiring nutrients to provide the energy needed to reach their target mucosal niche.

The colonic mucus layer is likely key to this adaptation, as the mucus is the largest source of endogenous nutrients within the mammalian colon (12, 13). The mucus is predominantly composed of the mucin (Muc2), forming into a sterile compact inner mucus layer and a detached outer mucus harboring mucus-dwelling bacteria (14). Muc2 is heavily *O*-glycosylated (15, 16), with the terminal position of these *O*-glycans frequently occupied by the monosaccharide sialic acid (Neu5Ac), which can be cleaved and released by an array of microbial sialidases (17). Several studies have demonstrated that the catabolism of sialic acid can affect the growth and composition of commensal gut bacteria (18–20). It also contributes to bacterial overgrowth in the gut when the host is exposed to certain chemicals or antibiotics. For example, dextran sodium sulfate-induced colitis was shown to cause an increase in sialidase activity by the resident microbiota, leading to increased availability of sialic acid and the outgrowth of commensal *E. coli* species (21). It was also demonstrated that the dramatic postantibiotic expansion of *Salmonella enterica* serotype Typhimurium and *Clostridium difficile* observed in infected mice involves the catabolism of microbiota-liberated sialic acid by these pathogens (22).

The above studies indicate that prior exposure to antibiotics or colitis-inducing chemicals may be a prerequisite to increase the availability of sialic acid, thereby promoting the expansion of enteric pathogens. However, it is unclear whether other enteric pathogens (such as *C. rodentium*) can use sialic acid to expand and/or promote its pathogenesis even in the absence of chemical/antibiotic-driven microbiota disruption. A previous study showed that *C. rodentium* was unable to grow on polysaccharides including mucin *O*-glycans (23), corresponding with its limited expression of mucin-degrading glycosyl hydrolases (24, 25). However, since *C. rodentium* can rapidly grow when given simple sugars (23), we investigated whether sialic acid metabolism contributes to its fitness and/or virulence. *C. rodentium* was found to both sense and migrate toward sialic acid, using the sialic acid transporter NanT, and was capable of using the monosaccharide as a sole carbon source for growth. Moreover, sialic acid strongly induced *C. rodentium*’s secretion of the autotransporter proteins Pic and EspC, again in a NanT dependent manner. These virulence factors significantly enhanced *C. rodentium*’s ability to degrade mucins, as well as adhere to IEC respectively, and correspondingly, a strain unable to sense/import sialic acid (*ΔnanT*) was significantly impaired in its ability to colonize the intestines of mice. We thus demonstrate that mucus-derived sialic acid functions as an important nutrient and as a key signal for an A/E bacterial pathogen to escape its luminal niche and successfully infect its host’s intestinal epithelium.

## Results

### *C. rodentium* Colonizes the Mucus Layer and Catabolizes Sialic Acid but Not Whole Mucins.

**Fig. 1.**
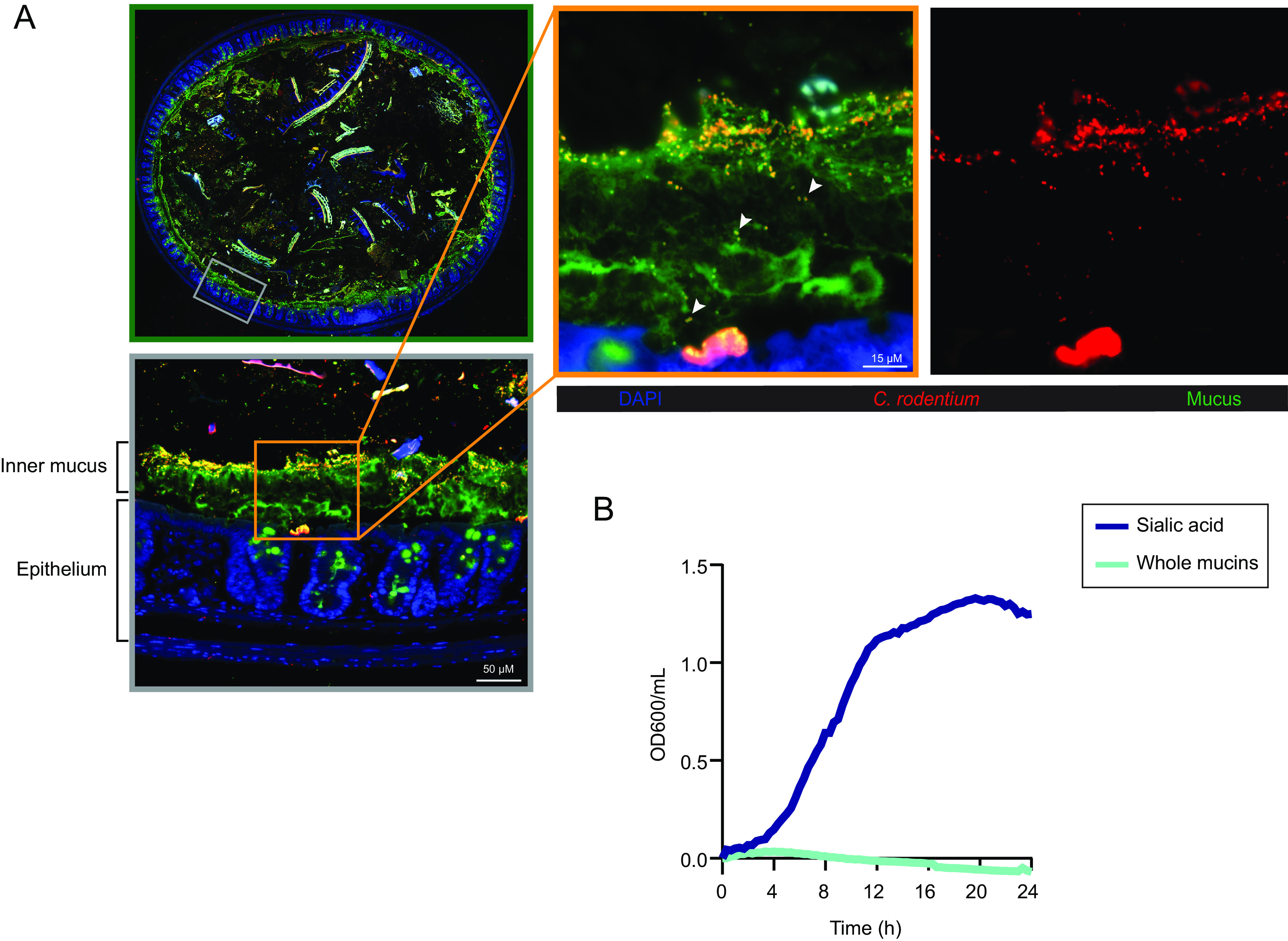
*Citrobacter rodentium* resides within the colonic mucus and catabolizes sialic acid. (*A*) *C. rodentium* localizes to the mucus layer. Representative immunofluorescence staining of mouse colonic tissue infected with *C. rodentium*. A colon cross-section (green panel) was stained with DAPI to detect DNA (blue), anti-*C. rodentium* (red) to visualize *C. rodentium,* and anti-Muc2 to visualize mucus (green). The gray panel is the enlarged view of the boxed region within the cross-section, original magnification = 200×. The orange panel is a magnified image indicating a subpopulation of *C. rodentium* localized to the inner mucus and traversing the mucus (arrowheads), with a separate image showing *C. rodentium* staining independently (red channel), original magnification = 630×. (Scale bar, 15 μm.) (*B*) *C. rodentium* uses sialic acid as a sole carbon source for growth. *C. rodentium* growth was measured by optical density (OD_600_) at 20-min intervals over 24 h at 37 °C in M9 minimal medium supplemented with 0.2% N-acetylneuraminic acid (sialic acid) or purified mucins. Data are presented as averages of cell growth (*n* = 9) from three independent experiments.

To define the biogeography of *C. rodentium* in vivo, in relationship to colonic mucus, we collected colonic tissues from mice at 6 days post *C. rodentium* infection (DPI) and costained them with antibodies recognizing *C. rodentium* and the mucin Muc2. As shown in Fig. 1*A*, populations of *C. rodentium* were found heavily colonizing the outer mucus layer or adherent to the colonic epithelium. In particular, a smaller subpopulation of *C. rodentium* was localized to the inner colonic mucus layer, in some cases appearing to traverse this barrier (arrowheads) (Fig. 1*A*).

Such close association between these *C. rodentium* populations and mucus led us to hypothesize that *C. rodentium* may use mucus and/or mucus-derived sialic acid as nutrient sources. To test this, we cultured *C. rodentium* in minimal medium supplemented with either whole mucins or with sialic acid. As shown in Fig. 1 *B* and *C**. rodentium* grew well in media containing sialic acid but failed to grow in media containing whole mucins. These data suggest that *C. rodentium* is able to use sialic acid as a sole carbon source for growth in vitro. However, as *C. rodentium* lacks the glycosidase enzymes necessary to cleave complex glycans from mucus (24, 25), it is unable to use whole mucins as a nutrient source.

### Sialic Acid Is Widely Expressed in the Colon at Baseline and during Infection.

**Fig. 2.**
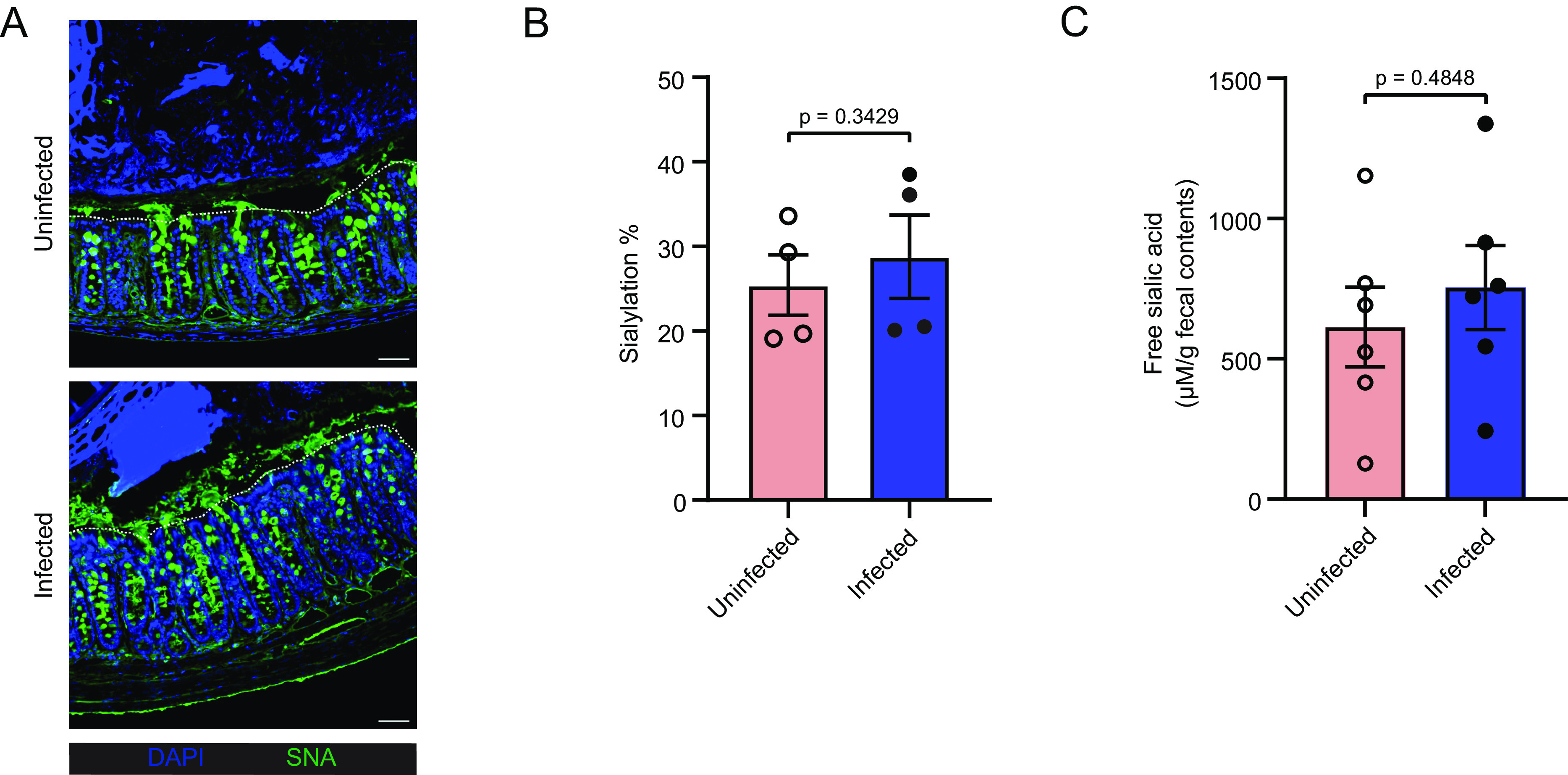
Sialic acid in the colon is mainly derived from mucus produced by goblet cells and widely expressed before and during *C. rodentium* infection. (*A*) Representative immunofluorescence staining of sialic acid on murine colonic sections with and without *C. rodentium* infection. Sections were stained with DAPI to detect DNA (blue) and SNA lectin (α2,6-sialic acid binding) to visualize sialic acid. Dotted lines indicate the apical side of the epithelium. Original magnification = 200×. (Scale bar, 50 µm.) (*B*) Degree of sialylation on Muc2 *O*-glycans of colonic mucus with (*n* = 4) and without (*n* = 4) infection with *C. rodentium* for 6 d. Released *O*-glycans from distal intestine were analyzed on PGC-LC-MS/MS. (*C*) Levels of free sialic acid in fecal contents of mice without (*n* = 6) and with (*n* = 6) *C. rodentium* infection for 6 d. All data are shown as mean ± SEM. Statistical significance calculated by the Mann–Whitney *U* test (*B* and *C*).

To evaluate the availability of sialic acid in the mouse colon, we stained colonic tissues with the *Sambucus nigra* agglutinin lectin (SNA) that binds to α2,6-sialic acid (26, 27). We found widespread α2,6-sialic acid staining in colonic goblet cells in both uninfected mice and mice at 6 DPI (Fig. 2*A*). We also examined the degree of sialylation, i.e., the covalent addition of sialic acid to the terminal end of glycoproteins (16, 28) in secreted mucus, through the analysis of *O*-linked glycan profiles with liquid chromatography–tandem mass spectrometry (LC-MS/MS). As shown in Fig. 2*B*, similarly high levels of sialylated glycans were detected in colonic mucus from both uninfected and 6 DPI mice. Sialylated mucus glycans can be cleaved by bacterial sialidases produced by some members of the gut microbiota, resulting in the release of free sialic acid that becomes accessible to other microbes, including invading pathogens (17, 29). We quantified the levels of free sialic acids in the fecal contents and detected similar levels under both uninfected and infected conditions (Fig. 2*C*). These findings indicate that sialic acid is abundant in the colon under both uninfected and infected conditions, but *C. rodentium* infection does not cause a significant change in its availability.

### Sialic Acid Is Used by *C. rodentium* as Both a Nutrient for Growth and Signal for Migration via the Transporter NanT.

**Fig. 3.**
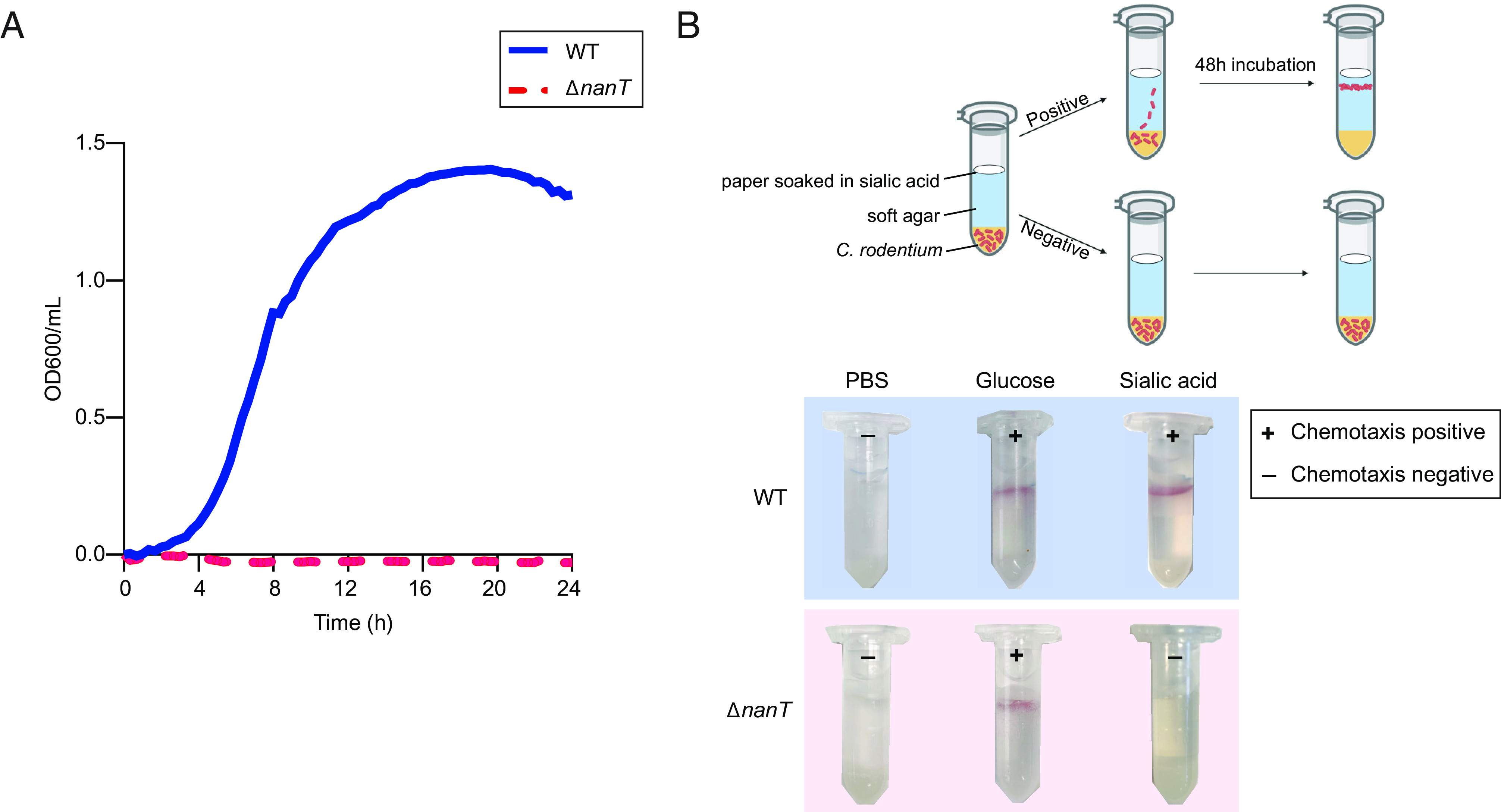
NanT is required for *C. rodentium* metabolism of sialic acid and *C. rodentium* chemotaxis toward sialic acid. (*A*) Growth analysis of Δ*nanT C. rodentium* in M9 minimal medium supplemented with 0.2% sialic acid in comparison to WT. Cultures were tracked with OD_600_ readings at 20-min intervals over 24 h at 37 °C. Data are presented as averages of cell growth (*n* = 9) from three independent experiments. (*B*) Migration of WT and Δ*nanT C. rodentium* toward sialic acid tested with a qualitative chemotaxis assay shown in schematic (*Top*). The “+” and “−” signs indicate the presence or absence of migration toward the stimulants, visualized through the formation of red rings of bacterial cells stained with 0.01% TTC (2,3,5-triphenyltetrazolium chloride). Images are representative of at least three independent experiments.

NanT is the predominant sialic acid transporter in most gram-negative bacteria (30–32). Although sialic acid uptake in *C. rodentium* has not been previously characterized, we found that the genome of *C. rodentium* contained the *nan* operon responsible for sialic acid catabolism, including the *nanT* (N-acetylneuraminic transporter) gene (24). To examine whether the *nan* operon was functional, we generated an in-frame deletion mutant of the *nanT* gene in *C. rodentium*, termed the Δ*nanT* strain. This mutant failed to grow in minimal media containing sialic acid (Fig. 3*A*), as compared to the rapid growth seen by wild-type (WT) *C. rodentium*, suggesting that *C. rodentium* is able to utilize sialic acid as a sole carbon source via the *nan* operon. This Δ*nanT* mutant did not display any growth defects when provided with other carbon sources (*SI Appendix*, Fig. S1).

Next, we examined whether *C. rodentium* was able to sense and actively migrate toward sialic acid. We developed an Eppendorf tube–based chemotaxis assay adapted from the study of *Campylobacter jejuni* chemotaxis (33). In this assay, the bacteria underwent upward directional movement through a layer of soft agar when exposed to a favorable chemoattractant placed at the top of the tube (Fig. 3*B*). We found that WT *C. rodentium* migrated toward sialic acid or glucose, but not PBS (control), as indicated by the red positive staining of triphenyltetrazolium chloride (TTC) near the top of the tube (Fig. 3*B*). In contrast, Δ*nanT C. rodentium* failed to migrate toward sialic acid (Fig. 3*B*). Thus, sialic acid is not only a nutrient source for *C. rodentium*, but its sensing by the transporter NanT also leads to *C. rodentium* migration toward the source of sialic acid.

### The Sialic Acid Transporter NanT Is Required for *C. rodentium* Colonization and Expansion in the Large Intestine.

**Fig. 4.**
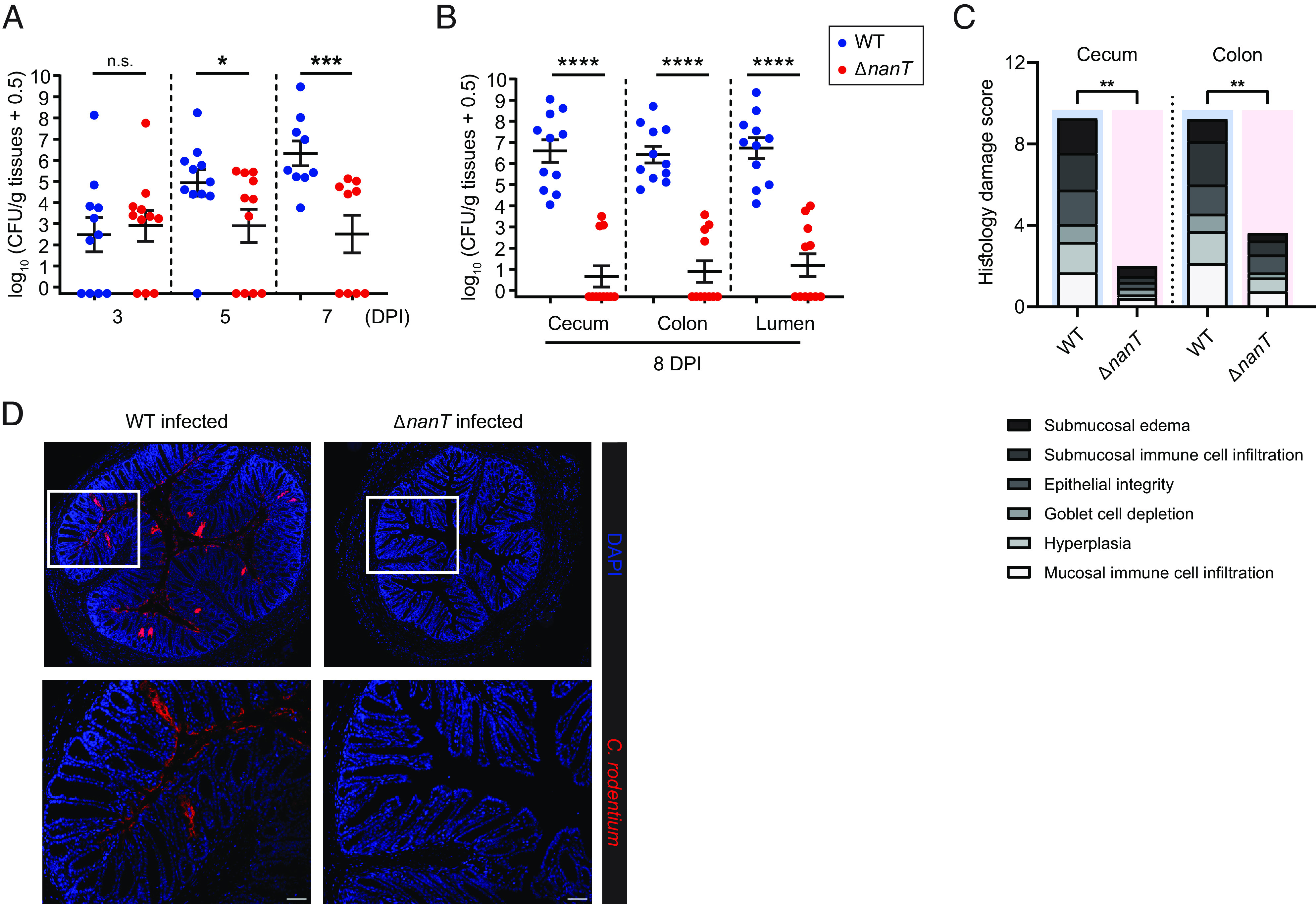
Δ*nanT C. rodentium* is significantly impaired in its ability to colonize mice. C57BL/6 mice were orally infected with 1 × 10^7^ CFU of WT (*n* = 11) or Δ*nanT* (*n* = 11) *C. rodentium* (*n*, number of biological replicates), and (*A*) stools were collected at 3, 5, and 7 days post infection (DPI); (*B*) intestinal tissues and luminal contents were collected at 8 DPI, plated, and enumerated for *C. rodentium* CFU. (*C*) Blinded histopathological scores of H&E tissue sections of mice infected with WT (*n* = 8) or Δ*nanT* (*n* = 8) *C. rodentium* (see *Materials and Methods* for scoring criteria). Means are indicated. Agreement among raters ensured by Kendall’s coefficient of concordance Wt = 0.848. (*D*) Representative colonic immunostaining for *Citrobacter* LPS (red) and DAPI (blue), showing little to no *C. rodentium* present on the Δ*nanT*-infected colon. *Lower* panels are expanded images of corresponding boxed regions in panels above. Original magnification = 200×. (Scale bar, 50 µm.) Data are represented as mean ± SEM from four independent experiments. ****P < 0.0001, ***P < 0.001, **P < 0.01, and *P < 0.05. Statistical significance calculated by the Mann–Whitney *U* test (*A*–*C*).

To address the in vivo role of sialic acid metabolism in *C. rodentium* pathogenesis, we gavaged C57BL/6 mice with either WT *C. rodentium* or Δ*nanT C. rodentium* and monitored pathogen shedding in the stool. In the early stages of infection (3 DPI), WT and Δ*nanT C. rodentium* were found to colonize the mouse GI tract at similarly low levels (Fig. 4*A* and *SI Appendix*, Fig. S2*A*). Over the following days, we observed a rapid expansion of WT *C. rodentium* from ~10^3^ CFU (colony-formingunit)/g (at 3 DPI) to a level of ~10^7^ CFU/g at 7 DPI. In contrast, the Δ*nanT C. rodentium* strain appeared unable to expand over this time, remaining at lower levels (at 7 DPI), similar to that seen at 3 DPI (Fig. 4*A*).

In the absence of an expansion of Δ*nanT*, we quantified pathogen burdens with dissected tissues at 8 DPI. Large numbers of WT *C. rodentium* were recovered from cecal and colonic tissues as well as from the luminal contents of infected mice. In contrast, Δ*nanT C. rodentium* was either completely cleared by 8 DPI or otherwise remained at very low numbers (<10^4^ CFU/g) (Fig. 4*B*). We also tracked the colonization dynamics of Δ*nanT* in individual mice by enumerating their stool burdens throughout the infection. While the Δ*nanT* mutant was able to colonize the intestines of all mice, the timing of its colonization and clearance varied (*SI Appendix*, Fig. S2*B*). Such rapid clearance exhibited in Δ*nanT-*infected mice suggests that the Δ*nanT* mutant is not only severely impaired in expanding its niche within the murine colon but in many cases unable to maintain its niche in the absence of a functional sialic acid utilization pathway.

We then analyzed pathological changes in colonic tissues from WT *C. rodentium*– and Δ*nanT C. rodentium*–infected mice. WT *C. rodentium* infection led to the disruption of colonic crypt architecture and extensive inflammatory cell infiltration. In contrast, the transient colonization by Δ*nanT C. rodentium* failed to induce an overt inflammatory response (*SI Appendix*, Fig. S3*A*), causing very modest colonic pathology (Fig. 4*C* and *SI Appendix*, Fig. S3*B*). In line with these findings, WT *C. rodentium* was found to heavily colonize the colonic mucosal surface (8 DPI), with many bacteria found intimately attached to the colonic epithelium, whereas Δ*nanT C. rodentium* were too few in numbers to be detected by immunostaining (Fig. 4*D*). Interestingly, exogenous administration of free sialic acid accelerated the colonization of *C. rodentium* (*SI Appendix*, Fig. S4). Thus, the sialic acid transporter NanT is required for *C. rodentium* colonization and expansion in the large intestine, likely through the import of sialic acid.

### Sialic Acid Enhances *C. rodentium’s* Ability to Degrade Mucins.

**Fig. 5.**
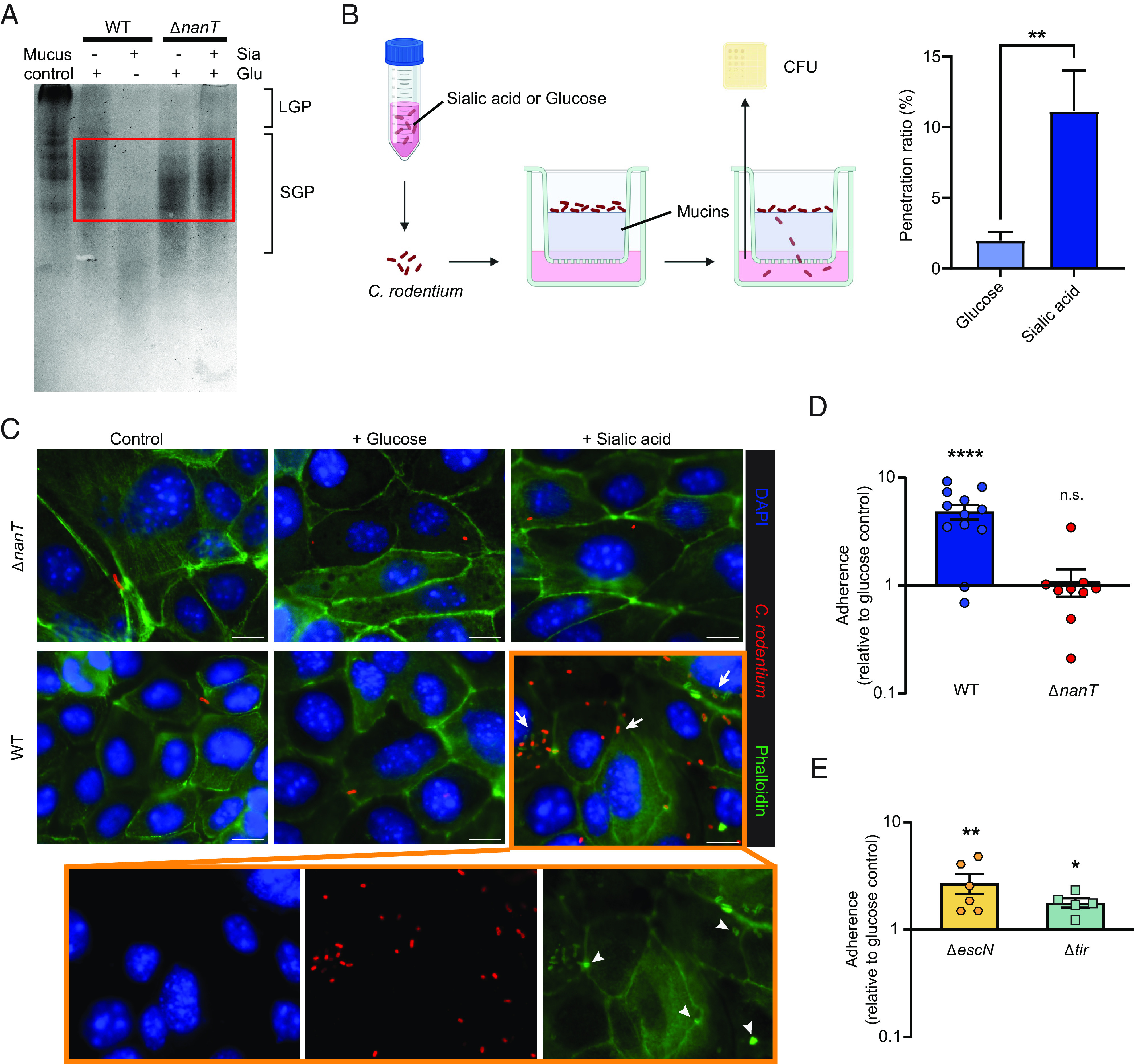
Sialic acid promotes *C. rodentium* mucin penetration and epithelial adherence. (*A*) Characterization of mucinolytic activity in secreted proteins of WT and Δ*nanT C. rodentium* cultured in sialic acid compared to the glucose controls. Proteins secreted from WT cultured in glucose or from Δ*nanT* demonstrated moderate mucinolytic activity, indicated by the clearance of large glycoproteins (LGP) in the stacking region of the gel and increased abundance of smaller glycosylated proteins (SGP, boxed area). Secreted proteins collected from WT sialic acid culture demonstrated enhanced mucinolytic activity, cleaving the mucin proteins into significantly lower molecular weights. The image is representative of four independent experiments. (*B*) Penetration of WT *C. rodentium* through the mucin layer when precultured in sialic acid compared to glucose. *C. rodentium* precultured in Dulbecco's modified Eagle medium (DMEM) with glucose or sialic acid was added to the top of purified mucins layered in the insert of a Transwell. DMEM medium was placed in the lower chamber and collected after 1 h incubation at 37 °C. Bacteria that penetrated the transwells were collected from the lower chamber and plated for CFU. The penetration ratio represents the percentage of bacteria that have penetrated the mucin layer. Data are shown in mean ± SEM from four independent experiments. (*C*) CMT-93 cells were infected with *C*. *rodentium* WT or Δ*nanT* in the presence or absence of sialic acid for 5 h and then washed to remove nonadherent bacteria and stained with phalloidin (green), anti-*C*. *rodentium* LPS (red), and DAPI to detect DNA (blue). The orange panel shows individual channels for WT *C*. *rodentium* infection in the presence of sialic acid. Arrows and arrowheads indicate increased adherence and pedestal formation respectively. Original magnification = 630×. (Scale bar, 10 μm.) (*D*) Adherence of *C*. *rodentium* WT or Δ*nanT* to CMT-93 cells. (*E*) Adherence of *C*. *rodentium* Δ*escN* or Δ*tir* (T3SS-deficient) strains to CMT-93 cells. Data represent relative fold changes in adherence of each *C*. *rodentium* strain to CMT-93 cells treated with sialic acid in comparison to glucose (*C* and *D*). Mean and SEM from three independent experiments are indicated. ****P < 0.0001, **P < 0.01, *P < 0.05, and n.s. = not significant. Significance levels were calculated by the Mann–Whitney *U* test (*B*, *D*, and *E*).

Since *C. rodentium* residing in the colonic lumen or outer mucus layer must penetrate the normally impenetrable inner mucus layer to infect the underlying epithelium, we examined whether sialic acid would impact *C. rodentium*’s ability to degrade mucins. *C. rodentium* was cultured in Dulbecco’s modified Eagle media (DMEM) supplemented with either sialic acid or glucose, with the supernatants collected and concentrated, followed by incubation with purified bovine submaxillary mucins (BSMs). After incubation, the BSM was run on a sodium dodecyl sulfate–polyacrylamide gel electrophoresis (SDS-PAGE) gel and stained by periodic acid–Schiff. As shown in Fig. 5*A*, in the lane loaded with untreated BSM (mucus control), the majority of staining is seen at the loading site and consists of large undigested glycoproteins (LGP), whereas smaller digested glycoproteins (SGPs) are seen to have migrated further down the lane. When the BSMs were incubated with supernatant from WT *C. rodentium* grown in glucose, the LGP band disappeared, leaving only the SGP bands (red boxed area), indicating that *C. rodentium* was able to partially degrade BSM, as previously reported (34). Notably, when the BSMs were incubated with the supernatant from sialic acid–treated WT *C. rodentium*, both the LGP and SGP populations largely disappeared, suggesting that sialic acid robustly increased the ability of *C. rodentium* to degrade mucins. Further, the ability of sialic acid to enhance mucin degradation is mediated by NanT, as BSM incubated with supernatants from sialic acid cultured Δ*nanT C. rodentium* showed only partial mucin degradation, similar to the profile seen without sialic acid (red boxed area).

To determine whether the enhanced mucinolytic activity induced by sialic acid in WT *C. rodentium* would accelerate the pathogen’s penetration through a mucin layer, we employed a mucin transmigration assay (35), in which *C. rodentium*, previously cultured in DMEM with glucose or sialic acid, was added onto mucins layered in Transwell inserts (3-µm pores, 6.5-mm-diameter, Corning), followed by an incubation at 37 °C for 1 h before enumerating bacteria that passed through the mucin layer and reached the lower chamber (Fig. 5*B*). While only 2% of *C. rodentium* grown in glucose-containing media were able to penetrate through the mucin layer within 1 h, preincubation of *C. rodentium* in sialic acid led to a significant fivefold increase (11%) in migration through the mucin layer (Fig. 5*B*). These findings demonstrate that exposure to sialic acid not only promotes *C. rodentium*’s mucin degradation but also facilitates its ability to penetrate and transit across mucins.

### Sialic Acid Enhances *C. rodentium’s* Ability to Adhere to IEC.

Next, we explored whether sialic acid impacted *C. rodentium*’s ability to adhere to IEC by performing an in vitro adherence assay with the CMT-93 murine IEC line. During an infection in DMEM with or without glucose, both WT and Δ*nanT C. rodentium* displayed similarly low levels of adherence. In contrast, when sialic acid was added and served as the sole carbon source in the media, WT *C. rodentium* showed significantly increased adherence to the IEC (arrows), with evidence of actin “pedestals” (arrowheads) beneath the majority of the adherent bacteria (a typical feature of A/E pathogen infection) (36). No increase in adherence was noted with the Δ*nanT* strain, however, as it remained at the same adherence level when exposed to sialic acid compared to glucose (Fig. 5 *C* and *D*). These data demonstrate that sialic acid not only promotes the degradation and penetration of mucus by *C. rodentium* but also the adherence of *C. rodentium* to IEC.

We also examined whether sialic acid’s promotion of adherence to IEC by *C. rodentium* was T3SS -dependent. Intimate attachment of A/E pathogens typically relies on EscN, an ATPase required for T3SS function, as well as the T3SS effector Tir (9). We therefore tested the effects of sialic acid on the ability of the Δ*escN* and Δ*tir C. rodentium* strains to adhere to CMT-93 IEC. Interestingly, while Δ*escN* and Δ*tir C. rodentium* had limited ability to intimately attach to IECs at baseline (with only 0.21% and 0.35% of the initial inoculum adhering to IECs, respectively), sialic acid still elevated their adhesion (Fig. 5*E*). These findings indicate that sialic acid’s ability to enhance *C. rodentium*’s adherence to IEC works even in the absence of a functional T3SS.

### Sialic Acid Induces *C. rodentium* to Secrete the Autotransporters Pic and EspC.

**Fig. 6.**
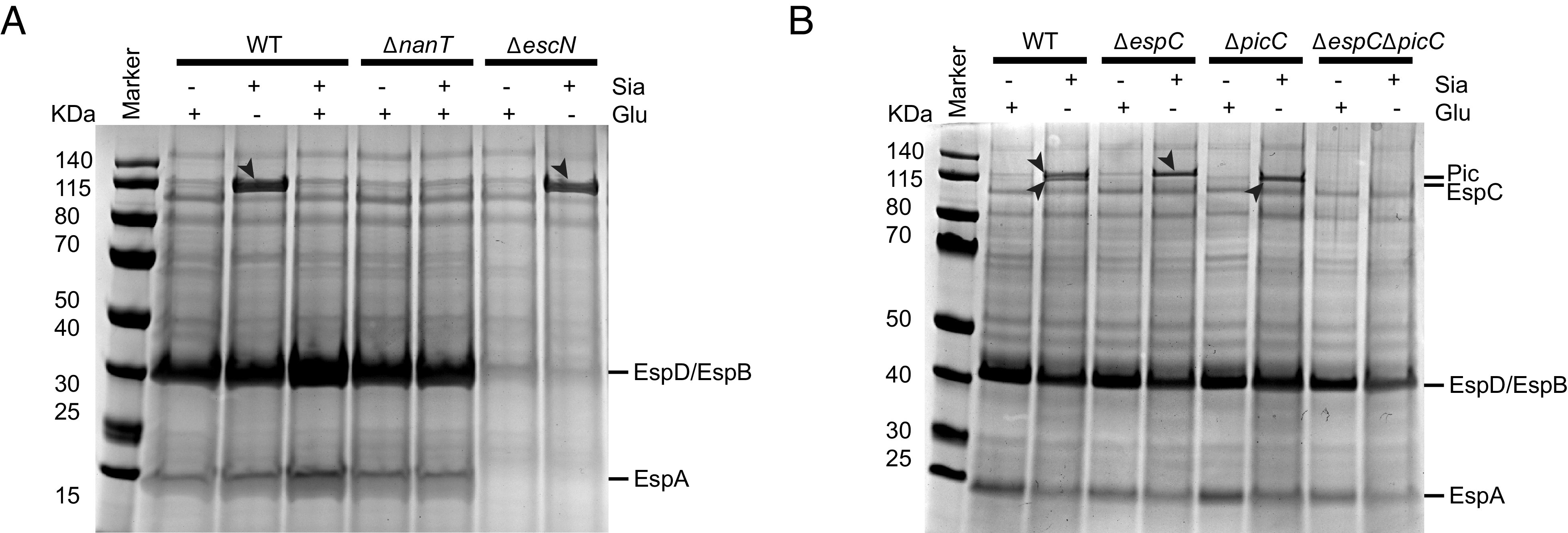
Sialic acid induces secretion of autotransporters Pic and EspC by *C*. *rodentium*. (*A*) Protein secretion profiles of WT, Δ*nanT,* and Δ*escN* *C*. *rodentium* after growth in DMEM with glucose or/and sialic acid as carbon sources. Δ*escN* is a negative control strain that is T3SS-deficient. (*B*) Protein secretion profiles of WT, Δ*espC,* Δ*picC,* Δ*espC*Δ*picC* *C*. *rodentium* after growth in DMEM with glucose or sialic acid. Secreted proteins in equal amounts of cultures for each strain (normalized by OD_600_) were analyzed in 4 to 12% SDS-PAGE and stained by Coomassie G-250. Arrowheads indicate proteins that are differentially secreted under sialic acid conditions.

To identify the proteins mediating the impact of sialic acid on *C. rodentium* pathogenesis, we cultured *C. rodentium* in DMEM in the absence/presence of sialic acid and examined the protein secretion profile. The addition of sialic acid did not appear to affect the secretion of the T3SS translocon proteins (i.e., EspA, EspB, and EspD) (Fig. 6*A*), which play a critical role in intimate adherence to IEC. The expression of *C. rodentium* T3SS is positively regulated by the master regulator Ler (37), so we also examined whether sialic acid impacted the expression of the T3SS using a bioluminescent reporter strain Cr-P_ler_*-lux*, in which WT *C. rodentium* carries a plasmid expressing the *lux*CDABE operon of *Photorhabdus luminescens* under the control of the *ler* promoter. Cr-P_ler_*-lux* showed comparable bioluminescence when grown in media containing sialic acid or media containing glucose (*SI Appendix*, Fig. S5). These findings suggest that sialic acid does not affect the expression or secretion of *C. rodentium*’s T3SS, the key machinery promoting cell adherence.

Intriguingly, there was a significant increase in the secretion of proteins of molecular weights close to 115 kDa (Fig. 6*A*). These protein bands were also present in sialic acid–containing cultures of the Δ*escN* *C. rodentium* strain that lacks a functional T3SS, suggesting that the secretion of these proteins is T3SS-independent (Fig. 6*A*). To identify these proteins, we took the supernatant of *C. rodentium* cultured in the presence of sialic acid and precipitated the proteins through the addition of 10% trichloroacetic acid as previously described (38), followed by analysis using an LC-MS/MS-based approach. As expected, many T3SS-secreted proteins were identified, such as EspD, EspB, EspA, and Tir (*SI Appendix*, Table S1). Notably, we identified two proteins, EspC (EPEC secreted protein C) and Pic (protease involved in intestinal colonization) that showed high abundance, with predicted molecular weights of 145.8 kDa and 141.0 kDa, respectively. To confirm the identity of these two proteins, we generated deletion mutants of *C. rodentium* [Δ*espC*, Δ*picC,* and Δ*espC*Δ*picC* (ΔΔ)] and analyzed their secretion profiles in the presence/absence of sialic acid. As shown in Fig. 6*B*, the deletion of *espC* did not affect sialic acid–induced secretion of Pic, and similarly, the deletion of *picC* did not affect sialic acid–induced secretion of EspC. When both *espC* and *pic* were deleted, the large protein band(s) close to 115 kDa typically induced by sialic acid were absent. Collectively, these data indicate that sialic acid induces the secretion of EspC and Pic by *C. rodentium*.

### Pic Mediates Sialic Acid–Enhanced Mucin Degradation by *C. rodentium*.

**Fig. 7.**
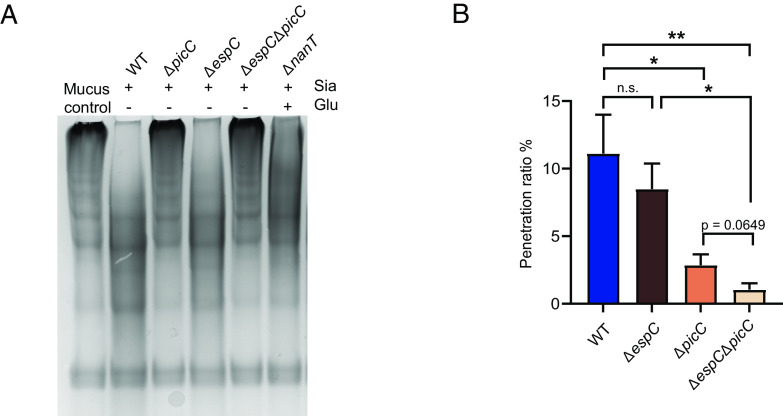
Sialic acid induces Pic mucinase in *C. rodentium* to accelerate degradation and penetration of the mucus layer. (*A*) Mucinolytic activity in secreted proteins of WT, Δ*picC,* Δ*espC,* Δ*espC*Δ*picC,* and Δ*nanT C. rodentium* after growth in sialic acid. Concentrated proteins were incubated with BSM (control) overnight. *C. rodentium* Δ*picC* and Δ*espC*Δ*picC* mutants are significantly impaired in degrading mucins. (*B*) Sialic acid’s induction of Pic enhances *C. rodentium*’s ability to penetrate mucins. *C. rodentium* WT, Δ*espC,* Δ*picC,* and Δ*espC*Δ*picC* mutants, after growth in sialic acid, were placed on top of Transwell inserts layered with purified mucins. Bacteria that penetrated the transwells were collected from the lower chamber and plated for CFU. Penetration ratio represents the percentage of bacteria that have penetrated the mucin layer after 1 h incubation at 37 °C. Data are shown in the mean ± SEM from four independent experiments. **P < 0.01, *P < 0.05, and n.s. = not significant. Significance levels were calculated by the one-way ANOVA.

Both EspC and Pic are proteases belonging to the family of serine protease autotransporters of the Enterobacteriaceae (SPATE) (39, 40). Notably, Pic expressed by enteroaggregative *E. coli* (EAEC) was previously shown to exhibit mucinolytic activity, potentially aiding the pathogen in penetrating intestinal mucus (41, 42). To address whether sialic acid enhances mucin degradation through the induction of Pic and/or EspC, we characterized the mucinolytic activities of the proteins secreted by WT *C. rodentium*, as well as the Δ*espC*, Δ*picC,* and ΔΔ strains when cultured in the presence of sialic acid. Notably, while exposure to sialic acid caused the expected increase in mucinolytic activity by the WT and Δ*espC* strains, it caused no increase in mucin degradation by the Δ*picC* or ΔΔ strains (Fig. 7*A*). We also examined the ability of these *C. rodentium* strains to transmigrate through a mucin layer after culturing them in sialic acid–containing media. Both Δ*picC* and the ΔΔ strain showed significant impairment in penetrating the mucin layer as compared to WT *C. rodentium* (Fig. 7*B*). While the ΔΔ strain showed a modestly reduced efficiency at transmigrating through the mucin layer as compared to Δ*picC* (Fig. 7*B*), the difference was not significant. These findings thus suggest that Pic plays the primary role in enhancing mucin degradation by *C. rodentium* following sialic acid stimulation, likely enhancing the ability of *C. rodentium* to penetrate the inner mucus layer overlying the colonic epithelium.

### Sialic Acid Enhances the Adherence of *C. rodentium* to IEC by Inducing EspC Secretion.

**Fig. 8.**
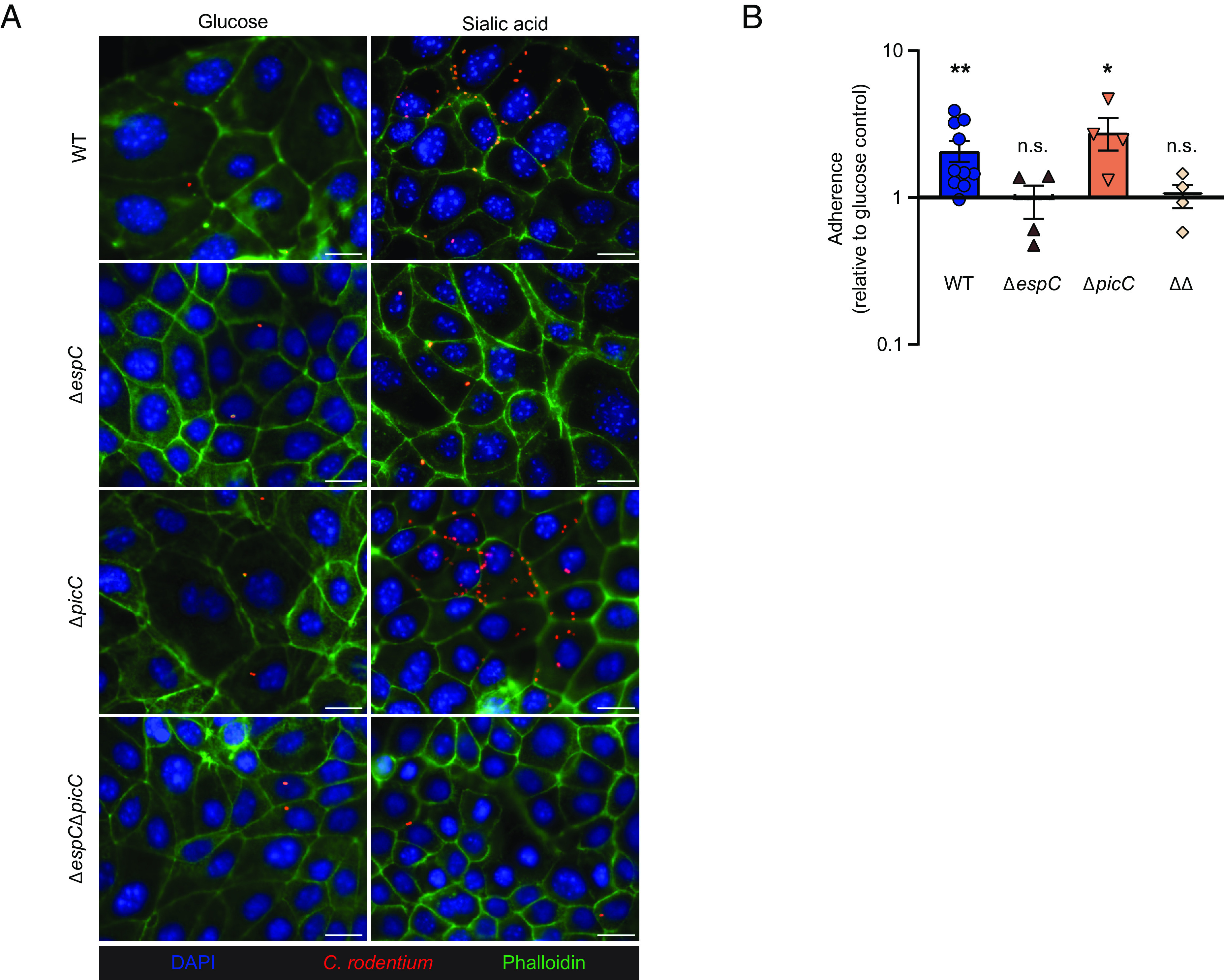
Sialic acid promotes *C. rodentium* adherence to epithelial cells through EspC. (*A*) CMT-93 cells were infected with *C*. *rodentium* WT, or Δ*espC,* Δ*picC,* Δ*espC*Δ*picC* mutants in the presence or absence of sialic acid for 5 h and then washed to remove nonadherent bacteria and stained with phalloidin (green), anti-*C*. *rodentium* LPS (red), and DAPI to detect DNA (blue). Original magnification = 630×. (Scale bar, 15 μm.) (*B*) Relative fold changes in adherence of *C. rodentium* WT, Δ*espC*, Δ*picC*, and Δ*espC*Δ*picC* (ΔΔ) mutants to CMT-93 cells treated with sialic acid in comparison to glucose. Data are shown in the mean ± SEM from two independent experiments. **P < 0.01, *P < 0.05, and n.s. = not significant. Significance levels were calculated by the Mann–Whitney *U* test.

Due to their highly up-regulated secretion by *C. rodentium* grown in sialic acid, we next asked whether either Pic or EspC contributed to the sialic acid–enhanced adherence of this pathogen to IEC. CMT-93 cells were infected with WT, Δ*espC*, Δ*picC,* or ΔΔ strains of *C. rodentium*, and the infected cells were either fixed and then stained (Fig. 8*A*) or homogenized with the bacteria plated and quantified as in Fig. 5*D*. Both WT and Δ*picC* showed increased adherence to IEC upon sialic acid treatment (Fig. 8 *A* and *B*). In contrast, the Δ*espC* and the ΔΔ strains showed remarkably lower levels of adherence, irrespective of the presence of sialic acid. These data suggest that sialic acid promotes the adherence of *C. rodentium* to IEC by inducing EspC secretion.

## Discussion

**Fig. 9.**
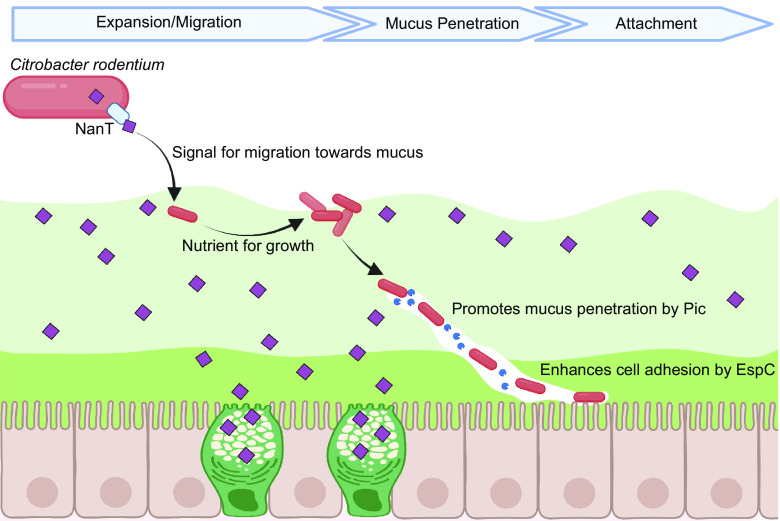
Sialic acid plays a key role in promoting *C. rodentium* pathogenesis within the gut. A proposed model illustrates the effect of sialic acid on *C. rodentium* pathogenesis. Sialic acid is captured from the environment, transported into the cell via *C. rodentium*’s transporter NanT and thereafter utilized as a growth substrate to promote the expansion of the pathogen within the gut. It also serves as a signal to direct the pathogen toward the mucus layer and specifically the mucins decorated with sialic acid. The metabolism of sialic acid further induces the secretion of Pic mucinase, thereby enhancing *C. rodentium*’s ability to degrade mucus and overcome this barrier. Upon traversing the mucus layer, sialic acid also induces the expression of the EspC protein that promotes *C. rodentium*’s adhesion to the colonic epithelial surface. Thus, sialic acid plays a pivotal role in licensing *C. rodentium*’s transition from the intestinal lumen to its mucosal adherent niche. Sialic acid is simplified in the illustration, representing both a monosaccharide form and a glycosylated form. The figure was generated with Biorender.com.

In this study, we demonstrate that the monosaccharide sialic acid plays a key role in *C. rodentium* pathogenesis within the mammalian gut, specifically in the poorly defined early stages of infection, and prior to A/E lesion formation on the intestinal mucosal surface. We showed that sialic acid is captured from the colonic environment via *C. rodentium*’s sialic acid transporter NanT and thereafter utilized as a growth substrate to expand within the gut. It also serves as a signal for *C. rodentium* migration toward mucus, and the sialic acid decorated mucins that comprise it. Sialic acid also induces the secretion of the Pic mucinase, enhancing *C. rodentium*’s ability to degrade mucins, facilitating its ability to traverse mucus barriers. This occurs concurrent with the increased expression of the EspC protein, which aids in *C. rodentium*’s initial adherence to the colonic epithelium (Fig. 9). Our study thus reveals intriguing insights into how an A/E bacterial pathogen escapes the colonic lumen and ultimately reaches the host’s epithelium.

The major impediment for luminal bacterial pathogens attempting to infect the colonic epithelium is the tightly adherent, largely impermeable and thus sterile “inner” mucus layer. Above this lies the loosely adherent “outer” mucus layer that extends into the lumen and is heavily populated by an array of commensal bacteria that feed upon the glycans found within (43). This includes most species of Proteobacteria, as they scavenge many of their nutrients, including mucin-derived sugars, from mucus-dwelling commensal bacteria specialized in degrading the outer mucus layer (1, 44). An inability to cross the inner mucus layer helps segregate these and other commensal microbes away from the host’s mucosal surface (43, 45). Thus, crossing this barrier is a necessary requirement for both mucosal adherent and invasive bacterial pathogens to cause disease (46), and correspondingly, mice lacking an intestinal mucus layer appear to undergo accelerated infections, although loss of mucus can lead to aberrant and/or attenuated pathogen interactions with the gut epithelium (47, 48).

The majority of research on A/E pathogens to date has focused on their T3SS (49), including what signals induce T3SS expression (37, 50, 51), as well as the ability of T3SS encoded effectors to subvert host cell functions (52, 53). In contrast, the pathogenic mechanisms employed by these pathogens to colonize their hosts and reach their target cells, prior to T3SS-mediated adherence, have received relatively little attention. While *C. rodentium* infection did not significantly alter the overall levels of free sialic acid within the host colon, its availability was a key limiting factor for pathogenesis, as provision of exogenous sialic acid accelerated the course of infection. Correspondingly, the Δ*nanT C. rodentium* strain proved dramatically attenuated. While capable of initially colonizing the mouse colonic lumen, the Δ*nanT* strain was unable to expand beyond a very modest level and in many cases was rapidly cleared from the colon. We suspect this reflects an inability of the mutant strain to acquire sufficient nutrients to fuel its passage across the mucus layer and reach the underlying epithelium.

It is intriguing that sialic acid strongly and specifically up-regulated the secretion of two members of the SPATE family of secreted serine proteases, namely Pic and EspC. SPATEs are large extracellular proteases, primarily secreted by disease-causing gram-negative bacteria (40). More than 25 SPATEs have been identified, with class 1 SPATEs shown to impact bacterial virulence through cytotoxic effects on IEC, whereas class 2 SPATEs appear to be largely immunomodulatory. Numerous in vitro studies have characterized the functions of those SPATES secreted by enteric pathogens such as *Shigella*, EPEC, EAEC, and uropathogenic *E. coli* (40). Unfortunately, due to the lack of relevant animal models for many of these pathogens, characterization of most SPATES has been limited to in vitro, in situ, or ex vivo conditions (54). Correspondingly, there is relatively little understanding of how these proteases interact with their hosts to promote disease or how their expression is regulated in vivo.

Our study determined that secretion of Pic by *C. rodentium* largely underlies the increased mucin degradation and mucin penetration demonstrated by *C. rodentium* following exposure to sialic acid. Most prior studies of Pic function have focused on the colonic human pathogen EAEC, with in vitro assays showing that Pic exhibits both mucinolytic and mucus secretagogue functions (41, 42, 55). We previously investigated whether Pic impacts *C. rodentium* pathogenesis in vivo but identified that loss of the Pic gene (*ΔpicC*) not only reduced mucinase activity but also led to abnormal colony morphology, increased adherence to other bacteria, as well as exaggerated activation of toll-like receptor 2 (34). Thus, defining the action of these autotransporters through null mutations may prove problematic, whereas defining their actions in response to stimuli (like sialic acid) that up-regulate their expression may offer a better approach to interrogate their roles in bacterial pathogenesis.

The other SPATE abundantly secreted by *C. rodentium* following exposure to sialic acid is EspC. EspC is expressed by a number of diarrheagenic *E. coli* pathotypes and has been shown to degrade a variety of substrates, including fodrin (56), a ubiquitous protein involved in actin polymerization. However, the effects of EspC on mucins require further assessment (39). Cell culture studies have shown that purified EspC displays enterotoxic effects on rat jejunal tissues (57), as well as cytotoxic effects when added to IEC in vitro (56), that were dependent on EspC’s protease activity. While studies with EPEC suggest that EspC is neither a T3SS effector nor required for A/E lesion formation (58), it does potentially interact with components of the T3SS (59, 60). EspC is one of several EPEC-derived proteins that insert into host cell membranes (61, 62), though EspC likely does this independently of the T3SS. In the current study, when compared to Pic, *C. rodentium*’s EspC played little role in the mucin degradation/penetration exhibited by *C. rodentium*. In contrast, its major role appears to be in promoting bacterial adherence to IEC, even in the absence of a functional T3SS. This could reflect its insertion into the host cell or, alternatively, the previously described ability of EspC to oligomerize into large “rope-like” structures that exhibit adhesive and cytopathic properties (63).

The expression of the *nan* operon in *E. coli* is known to be regulated by the transcriptional regulators cyclic AMP receptor protein (CRP) and NanR (64). In the presence of sialic acid and in the absence of glucose, the repressor NanR is inactivated, while CRP is activated, leading to increased expression of the *nan* operon. Similar to *E. coli*, the regulatory region of *C. rodentium*’s *nan* operon contains CRP and NanR binding sites (*SI Appendix*, Fig. S6), suggesting that the *nan* operon of *C. rodentium* and *E. coli* share a conserved regulatory mechanism. CRP binding motifs were also found (124 bp upstream of the start codon for *picC* and 123 bp for *espC*) in the regulatory regions of both the *picC* and *espC* genes in *C. rodentium*. In line with this, Pic and EspC are minimally secreted in WT *C. rodentium* in the presence of glucose compared to sialic acid (Fig. 6*B*). Since sialic acid induces the *nan* operon expression through NanR and CRP, we suspect *picC*/*espC* genes are also repressed by NanR. However, we were unable to identify any notable NanR operators (containing GGTATA repeats) (65) in the regulatory regions of *picC*/*espC*. This suggests that the upregulation of EspC and Pic secretion in response to sialic acid may involve additional regulatory mechanisms, which await further investigation.

In conclusion, sialic acid plays a key role in promoting *C. rodentium* pathogenesis within the mammalian gut, specifically regarding the poorly defined stages of virulence prior to A/E lesion formation on the intestinal mucosal surface. While studies have previously identified a role for sialic acid catabolism in the postantibiotic expansion of enteric bacterial pathogens (22), our study shows such a role in the absence of antibiotics. Our findings highlight the central role played by mucus and its components in the pathogenesis of *C. rodentium* and potentially other A/E pathogens. Moreover, the identification of key nutrients utilized by bacterial pathogens offers exciting potential for developing alternative antimicrobial approaches to conventional antibiotics.

## Materials and Methods

### Bacterial Strains and Culture Conditions.

*C. rodentium* strain DBS100 (streptomycin-resistant) was used as the WT bacterial strain in this study. Bacteria were routinely grown on Luria–Bertani (LB) agar plates or in LB broth with shaking (200 rpm) at 37 °C overnight. Where appropriate, streptomycin was supplemented at 100 μg/mL, and kanamycin was supplemented at 50 μg/mL.

### Mutant Construction.

In-frame deletion mutants of *C.* *rodentium* DBS100 were generated using overlap extension PCR (66) with a suicide vector pRE112 (67). Two PCR fragments, upstream and downstream of each target gene, respectively, were amplified using DNA extracted from the WT strain as a template by primers with flanking KpnI restriction sites or primers with flanking SacI restriction sites as detailed in *SI Appendix*, Table S2. The two PCR fragments, sharing an overlapping sequence, were used as the template for a secondary PCR, the product of which was then digested with KpnI and SacI restriction enzymes, and directly cloned into pRE112 (chloramphenicol resistant). The plasmid construct was transformed into *E. coli* SM10 λ pir via electroporation and introduced into the WT strain by conjugation. Double-cross-over mutants were selected by plating onto LB (no sodium chloride) agar plates containing 5% sucrose. The resulting mutants were confirmed by PCR and DNA sequencing with check-Forward and check-Reverse primers (*SI Appendix*, Table S2).

### *C. rodentium* Growth Curve.

Overnight bacterial cultures grown (shaking) in LB broth at 37 °C were pelleted by centrifugation, washed three times with M9 media, and resuspended in M9 media. Resuspended cultures were then diluted 1:100 in 200 μL M9 media supplemented with 0.2% N-acetylneuraminic acid (sialic acid, Carbosynth) or purified porcine stomach mucins (Sigma) into a sterile 96-well plate (Corning) and incubated at 37 °C with shaking for 24 h. The optical density at 600 nm (OD_600_) was taken every 20 min using a Varioskan LUX microplate reader (Thermo Fisher). Each experiment was performed with at least three biological replicates. The results were confirmed by measuring bacterial densities in CFU per ml of culture in parallel experiments run in test tubes.

### Murine Colonic Mucin Isolation, Purification, and Characterization.

The colonic mucus was gently scraped from uninfected C57BL/6 mice (control) and mice infected with *C. rodentium* at 6 DPI. The mucus was partially purified from mucosal scrapings by repeated extraction with 3 × 100 µL aliquots of 6 M guanidine hydrochloride (GuHCl) (68). The Muc2-containing insoluble fraction was washed with 80% ice cold acetone to remove excess GuHCl and centrifuged at 14,000 rcf for 10 min to obtain a Muc2-containing pellet. *O*-glycans were released in solution by reductive beta elimination by the addition of 100 µL of 1 M NaBH_4_ in 100 mM KOH and incubation at 50 °C for 16 h. The reaction was quenched with 10 µL glacial acetic acid and then desalted with Dowex AG-50W-X8 cation exchange resin and porous graphitized carbon (PGC) packed into 100 µL C18 OMIX tips (Agilent). Desalted *O*-linked glycans were analyzed by PGC-LC-MS/MS in negative ion mode (69). Glycan peak areas were processed with Skyline 3.7.0.

### Free Sialic Acid Quantification.

Mouse fecal samples were collected and snap-frozen before use. Feces were weighed and reconstituted in distilled water (200 mg/mL) and homogenized for 15 min at maximum speed. Clarified supernatants were obtained after centrifuge for 15 min at 14,000 × g and used to measure free sialic acid levels using the QuantiChrom Sialic Acid Assay Kit (BioAssay Systems) according to the manufacturer’s protocol.

### Chemotaxis Assay.

Bacterial chemotaxis assay was performed in Eppendorf tubes as previously described (33). In brief, 4 × 10^9^ CFU of *C. rodentium* were pelleted, resuspended in 500 μL PBS-based 0.4% agar, and transferred to the bottom of a 2 ml Eppendorf tube. Another 1 mL of PBS-based 0.4% agar containing no bacteria was layered on top of the cell suspension. A sterile piece of Whatman paper, soaked with 100 mM solution of glucose, sialic acid (Neu5Ac), or PBS was placed on top. Samples were incubated at 37 °C for 2 d. After incubation, 200 µl of 0.01% 2,3,5-TTC was added to visualize *C. rodentium* that migrated through the PBS-agar layer toward the compounds added to the Whatman paper. Positive results were presented as formation of red rings of bacterial cells near the top of the tubes stained by TTC after 4 h of incubation.

### Mouse Infections.

Sex-matched C57BL/6 mice (6 to 10 wk old), bred under specific pathogen-free conditions at BC Children’s Hospital Research Institute (BCCHRI), or purchased from Charles River Laboratories were used in this study. Mice were orally gavaged with 1 × 10^7^ ∼ 2.5 × 10^8^ CFU of *C. rodentium*. To monitor *C. rodentium* colonization, fecal pellets were collected, homogenized in PBS, and plated on LB agar containing streptomycin. At the end of each experiment, mice were anesthetized with isofluorane and euthanized by cervical dislocation. Colonic tissues were immediately fixed in 10% neutral buffered formalin (Fisher Scientific) for 24 h or in Methacarn fixative (60% methanol, 30% chloroform, and 10% glacial acetic acid) for 3 to 24 h at 4 °C. Pathogen burdens within tissues or luminal compartments were enumerated through serial dilutions on selective agar plates.

### Histopathological Scoring.

Histopathological analysis was performed on hematoxylin-eosin-stained (H&E) cecal and distal colon tissue sections. In brief, tissues previously fixed in 10% neutral buffered formalin were paraffin-embedded and sectioned at 5 µm. These sections were stained with H&E, photographed, and scored by two blinded observers using previously established criteria (70). Tissue sections were assessed for 1) submucosal edema (0: no change, 1: mild, 2: moderate, and 3: severe), 2) submucosal neutrophil and mononuclear cell infiltration (per 400× magnification field) (0: <5, 1: 5 to 20, 2: 21 to 60, 3: 61 to 100, and 4: >100 cells/field), 3) epithelial integrity (0: no pathological changes detectable, 1: epithelial desquamation (few cells sloughed, surface rippled), 2: erosion of epithelial surface (epithelial surface rippled, damaged), 3: epithelial surface severely disrupted/damaged, large amounts of cell sloughing, and 4: ulceration), 4) goblet cell depletion (0: no change, 1: mild depletion, 2: severe depletion, and 3: absence of goblet cells), 5) crypt hyperplasia (0: no change, 1: 1 to 50%, 2: 51 to 100%, and 3: >100%), and 6) mucosal mononuclear cell infiltration (per 400× magnification field) (0: no change, 1: <20, 2: 20 to 50, and 3: >50 cells/field). A maximum score under this scale is 20.

### In Vitro *C. rodentium* Adherence Assay.

CMT-93 (mouse rectal epithelial) cells (ATCC CCL-223) were seeded in 24-well plates at a density of 5 × 10^4^ cells/well and grown until reaching >90% confluence (37 °C, 5% CO_2_). Prior to infection, cells were washed twice with PBS and preincubated in DMEM supplemented with 2% fetal bovine serum (Life Technologies) with or without 0.2% sialic acid for 30 min. Cells were infected with an overnight culture of *C. rodentium* at an MOI of 50 for 4 to 5 h. After infection, the supernatants of infected cells were removed, and the cell monolayers were washed three times with PBS and subsequently treated with 200 μL of 0.1% Triton X-100 PBS for 5 min at room temperature to lyse the cells. Adherent bacteria were enumerated by serial dilutions in PBS and plated onto LB–streptomycin agar plates. The percentage of adhered bacteria was calculated by dividing the number of adhered bacteria by the number of total bacteria.

### Immunofluorescence and Lectin Staining.

Paraffin-embedded tissue sections (5 μm) were deparaffinized by heating at 60 °C for 8 min, cleared with xylene, and rehydrated with 100%, 95%, and 70% ethanol, followed by dH_2_O. Deparaffinized sections were boiled in sodium citrate buffer (pH 6.0) for 40 min, followed by 1 h blocking with blocking buffer (PBS containing 2% donkey serum, 0.1% Triton-X100, and 0.05% Tween 20). For visualizing *C. rodentium* localization in the mucus, methacarn-fixed mouse distal colons were stained with the following primary antibodies—rat anti-*C*. *rodentium* Tir (gift from W. Deng), rabbit anti-Muc2 (Boster), and rabbit anti-Muc2 (Novus), which were then probed with Alexa Fluor 488-conjugated donkey anti-rabbit IgG (Life Technologies) and Alexa Fluor 568–conjugated donkey anti-rat IgG (Life Technologies). To detect WT and Δ*nanT C. rodentium* in tissue sections, formalin-fixed colonic tissues were stained with antisera against *E. coli* monospecific O152 (rabbit polyclonal, SSI Diagnostica) that recognizes *C. rodentium* O-antigen (71, 72), and labeled with Alexa Fluor 568–conjugated donkey anti-rabbit IgG (Life Technologies). To detect the distribution of sialic acid, fluorescein conjugated–SNA (Vector laboratories) was used. Stained tissues were mounted using ProLong Gold Antifade reagent containing DAPI (Invitrogen).

To perform immunofluorescent staining on infected tissue culture cells, sterile coverslips were seeded and infected as described above. After postinfection washes in PBS, coverslips were fixed in 4% paraformaldehyde (Fisher Scientific) for 15 min, rinsed in PBS twice, and permeabilized with 0.1% Triton X-100 and 0.05% Tween in PBS for 15 min. Coverslips were stained with in *E. coli* monospecific O152 (rabbit polyclonal, SSI Diagnostica) for 1 h, followed by secondary antibody staining with anti-rabbit Alex Fluor 568 and Alexa Fluor 488 or 680-phalloidin for 1 h, washed, and mounted with ProLong Gold Antifade reagent containing DAPI (Invitrogen). Slides were viewed on a Zeiss AxioImager microscope, and images were taken using an AxioCam HRm camera operating through Zen software.

### Protein Secretion Analysis.

*C. rodentium* strains were grown overnight shaking in LB broth at 37 °C and subcultured 1:40 into Dulbecco’s modified Eagle’s medium (DMEM) with or without supplementation of 0.1% sialic acid to induce protein secretion in a tissue culture incubator with 5% CO_2_ at 37 °C until reaching midexponential-phase growth. Secreted proteins in the supernatant of equal amount of cultures (normalized by OD_600_) were obtained as previously described (38). Protein secretion was analyzed in 4 to 12% SDS-PAGE and stained with Coomassie G-250.

For proteomic analysis, protein pellets were reduced with dithiothreitol and alkylated with iodoacetamide, followed by enzymatic digestion. Proteins were identified through LC–MS/MS analysis with a search against *C. rodentium*’s protein sequence database.

### Mucinolytic Activity Assay.

Protein secretion was induced in DMEM with or without 0.1% sialic acid as described above.

Equal volumes of supernatants (normalized by OD_600_) were collected and filtered through a 0.22-µm filter to remove bacterial cells. Supernatants were then concentrated through Amicon Ultra 4 (50-kDa cutoff; Millipore) filters. Secreted protein concentrates (20 µL) were incubated overnight at 37 °C with 6.5 µL of 2% purified BSM (Sigma). Mucin degradation was analyzed on a 3 to 8% Tris–Acetate gel and visualized by staining with the Pierce glycoprotein staining kit (Thermo Scientific).

### Mucin Transmigration Assay.

Transwell filters (24-well insert, 3.0-µm pores, Corning) were coated with 100 µL of 30 mg/mL mucin and placed onto 24-well plates containing 250 µL DMEM in the bottom chambers. Next, 10 µL of *C. rodentium* induced in DMEM (5.0 × 10^6^ CFU) was added onto the top of the mucin layers and incubated at 37 °C for 1 h. Bacteria that were able to transmigrate to the bottom of the well were collected and enumerated through serial dilutions on LB-streptomycin agar plates. The percentage of *C. rodentium* crossing the mucus layers was normalized to control samples from Transwells uncoated with mucins.

### Ethics Statement.

All mouse experiments were performed according to the protocol A19-0254 approved by the University of British Columbia’s Animal Care Committee and in direct accordance with the Canadian Council on Animal Care guidelines.

## Supplementary Material

Appendix 01 (PDF)

## Data Availability

All study data are included in the article and/or *SI Appendix*.
