# Supplementary material for: Sialic acid plays a pivotal role in licensing Citrobacter rodentium’s transition from the intestinal lumen to a mucosal adherent niche
Source: Proc Natl Acad Sci U S A. 2023 Jul 3;120(28):e2301115120. doi: 10.1073/pnas.2301115120 (PMC10334811; doi:10.1073/pnas.2301115120)
Supplement: Supplementary file 1 — Appendix 01 (PDF) [file pnas.2301115120.sapp.pdf]

PNAS

Supporting Information for

Sialic acid plays a pivotal role in licencing *Citrobacter rodentium's* transition from the intestinal lumen to mucosal adherent niche

Qiaochu Liang[1], Caixia Ma[1], Shauna M. Crowley[1,2], Joannie M. Allaire[1,3], Xiao Han[1], Raymond W. W. Chong[4], Nicolle H. Packer[4], Hong Bing Yu[1*], and Bruce A. Vallance[1*]

*Hong Bing Yu, Bruce A. Vallance
Email: hby@mail.ubc.ca, bvallance@cw.bc

This PDF file includes:

Supporting text
Figures S1 to S6
Tables S1 to S3

Supplementary Materials and Methods

RNA extraction and quantitative real-time PCR for host cytokine responses

RNA from mouse cecal and distal colonic tissues was preserved in RNAlater (Qiagen) and extracted using the RNeasy Mini kit (Qiagen) according to the manufacturer's instructions. Total RNA was quantified using a NanoDrop spectrophotometer (Thermo Fisher). Complementary DNA (cDNA) was constructed from reverse transcription of 500 ng RNA using 5X All-In-One RT MasterMix (Applied Biological Materials) according to the manufacturer's instructions. The cDNA was then diluted 1:5 in RNase/DNase free H_2O and 5 µl was used for 20 µl quantitative PCR (qPCR) reactions that contained primers (300 nM) and 10 µl SsoFast EvaGreen Supermix (Bio-Rad). qPCR reactions were carried out using a Bio-Rad CFX connect Real-time PCR detection system, with the specificity for each of the PCR reactions confirmed by melting point analysis. The expression of genes was normalized to housekeeping gene, *Ribosomal Protein Lateral Stalk Subunit (Rplp0)*. mRNA transcript expression was normalized to the relative expression of the reference genes using the $2^{-(\Delta Ct)}$. For treatment conditions, mRNA transcript expression was normalized to the control group (untreated condition) using the $2^{-(\Delta\Delta Ct)}$ method and presented as relative expression values. Primers used for qPCR are listed in Table S3.

Detection of *ler* expression

Bioluminescent reporter strain P_{ler}-*lux C. rodentium* were diluted 1:40 in DMEM supplemented with 0.1% glucose or sialic acid from overnight LB cultures and grown in sterile 96-well black bottom microplates (Corning) for 5 h to late exponential phase under tissue culture conditions (20% O_2, 5% CO_2) at 37°C. Readings of luminescence and OD_{600} were taken with a Varioskan LUX microplate reader (Thermo Scientific) using the SkanIt software (Thermo Scientific) every 30 min for *ler* expression analysis.

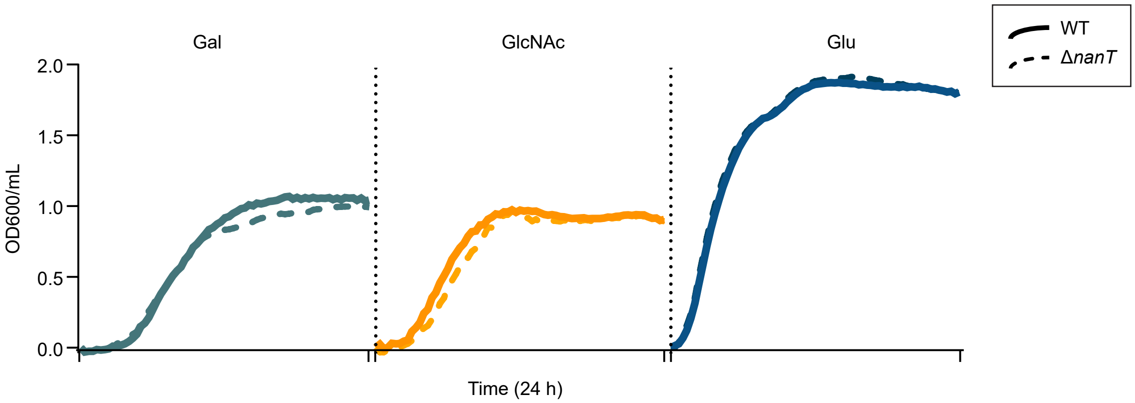

Fig. S1. Δ*nanT C. rodentium* does not display growth defects when provided other other mucin sugars or glucose. Growth analysis of WT and Δ*nanT C. rodentium* in M9 minimal medium supplemented with 0.2% galactose (Gal), N-acetylglucosamine (GlcNAc) or glucose (Glu). Cultures were tracked with OD_{600} readings at 20-minute intervals over 24 hours at 37°C. Data are presented as averages of cell growth (*n* =9) from three independent experiments.

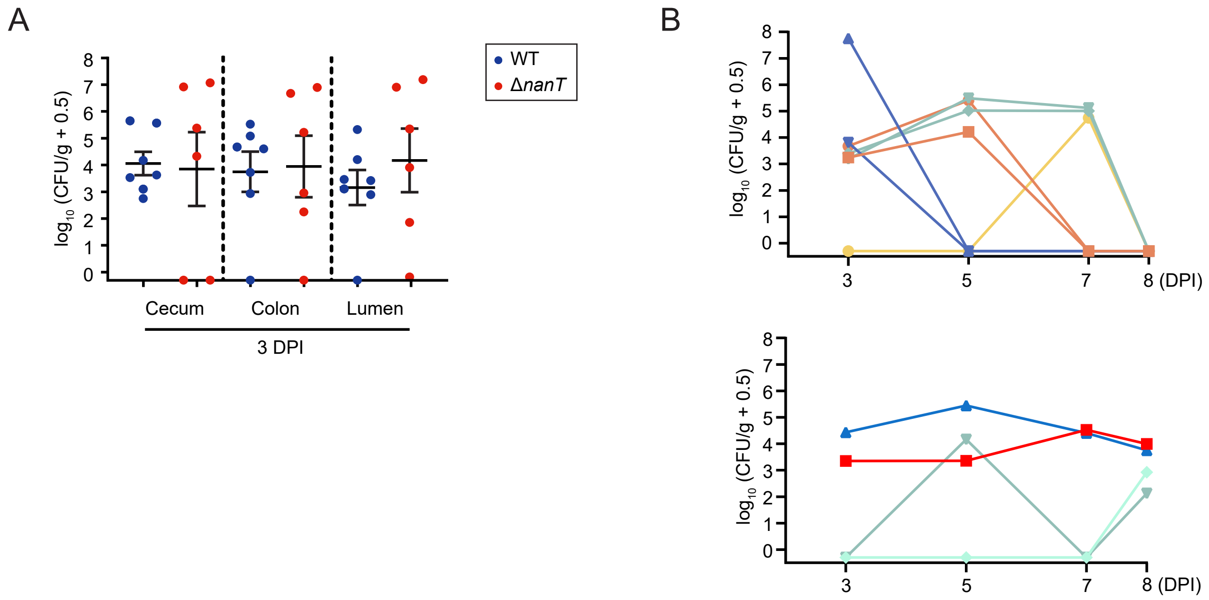

Fig. S2. Δ*nanT* is able to colonize the mouse GI tract at low levels at various timings. (A) Δ*nanT C. rodentium* colonizes the mouse intestines at similarly low levels as WT in the early stage of infection. C57BL/6 mice were orally infected with 1 × 10^7 CFU of WT (n = 7) or Δ*nanT* (n = 6) *C. rodentium* and intestinal tissues and luminal contents were collected at 3 days post infection, plated and enumerated for *C. rodentium* CFU. Data from two independent experiments were pooled. Mean and SEM are indicated. (B) Colonization of Δ*nanT* in each individual mouse, enumerated from stools collected on 3, 5 and 7 DPI, shows that Δ*nanT* was able to colonize the intestines of all mice, but the timing of colonization, and clearance varied. Most mice completely cleared by 8 DPI (top), while others remained at very low numbers (bottom).

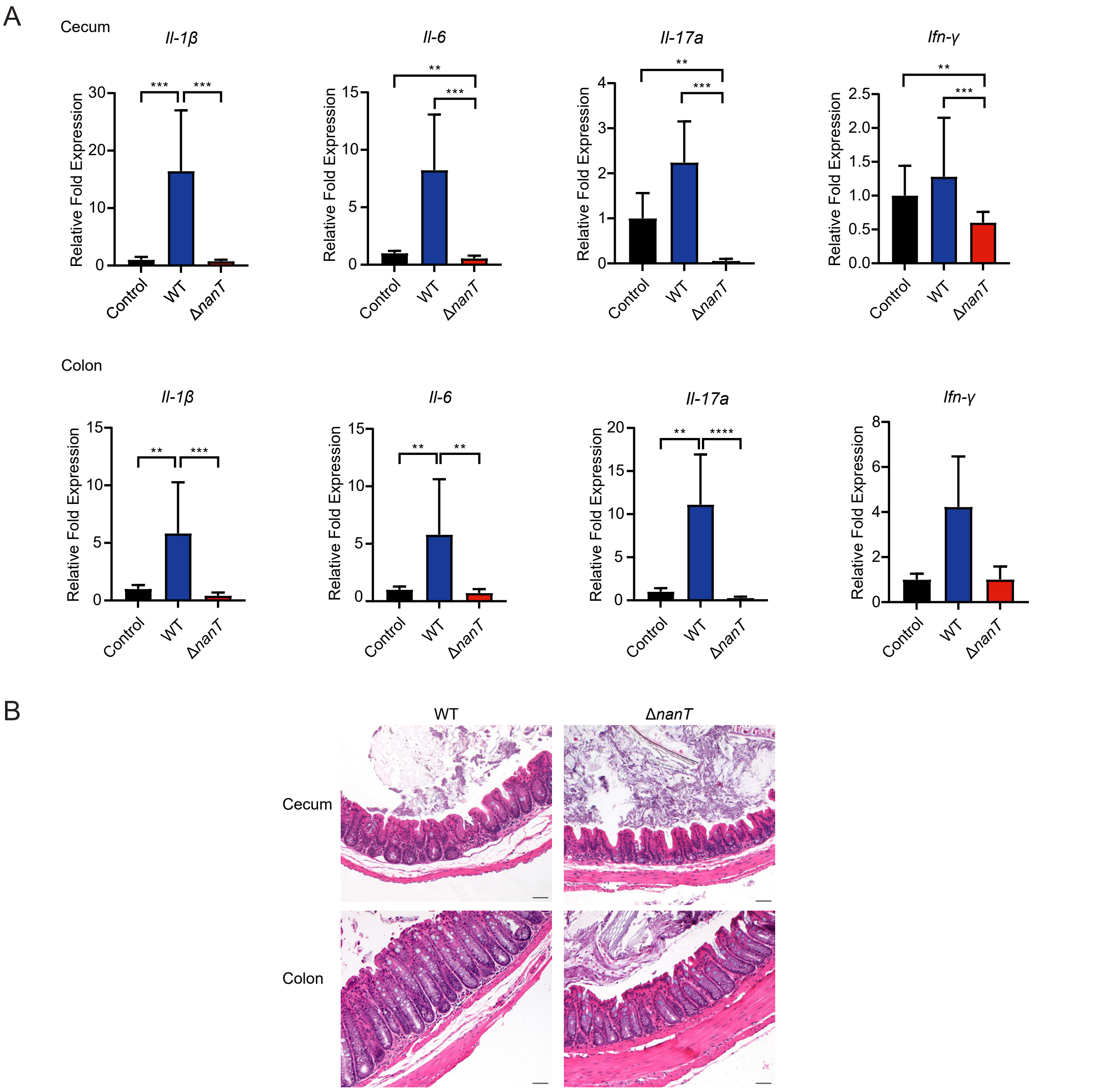

Fig. S3. *ΔnanT C. rodentium* failed to induce an overt inflammatory response. (A) qPCR analysis of inflammatory genes in mouse cecum and colon, expressed as fold change over control uninfected mice. ****$p < 0.0001$, *** $p < 0.001$, ** $p < 0.01$. Statistical significance calculated by one-way ANOVA. (B) Representative H&E-stained cecal and distal colonic sections from WT and Δ*nanT* infected mice. Original magnification = 200×. Scale bar = 50 μm.

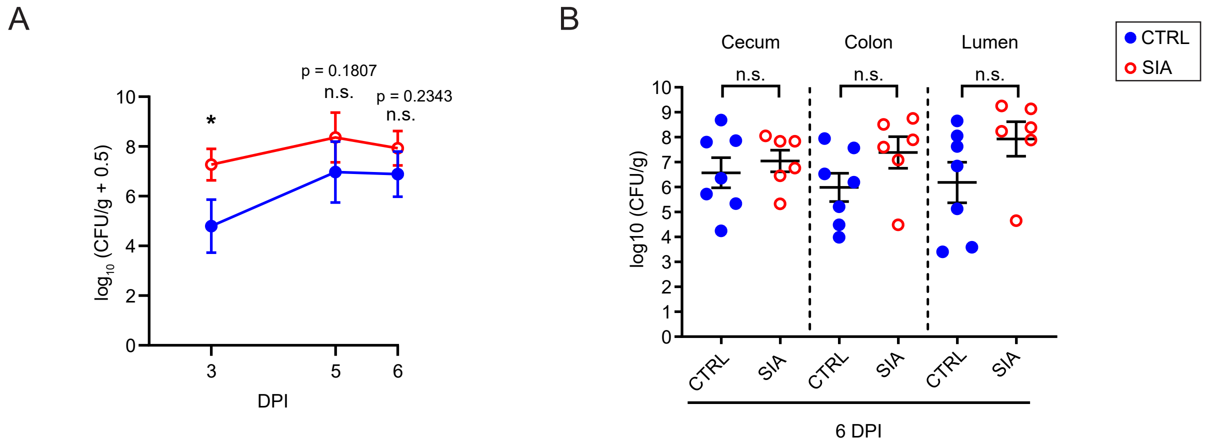

Fig. S4. Exogenous administration of free sialic acid accelerated *C. rodentium* colonization. *C. rodentium* burdens were compared between mice treated with normal drinking water (CTRL) or exogenous free sialic acid (SIA). Pathogen colonization was enumerated in (A) fecal contents collected at 3, 5, 6 (DPI) and (B) intestinal tissues and luminal contents collected at 6 DPI. Data are shown in the mean ± SEM from two independent experiments. * $p < 0.05$, n.s. = not significant. Significance levels calculated by Mann-Whitney U-test.

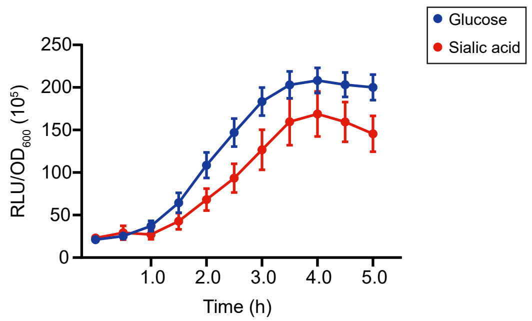

Figure S5. Expression of *ler* is not significantly altered in response to sialic acid compared to glucose. Bioluminescent reporter strain P_{ler}-*lux C. rodentium* was grown in DMEM with sialic acid or glucose under tissue culture conditions (20% O_2, 5% CO_2) for 5 hours. Expression of *ler* over time was measured by luciferase activity (relative luminescence units per optical density, RLU/OD_{600}), normalized to media containing no bacteria. Data are shown in the mean ± SEM from three independent experiments.

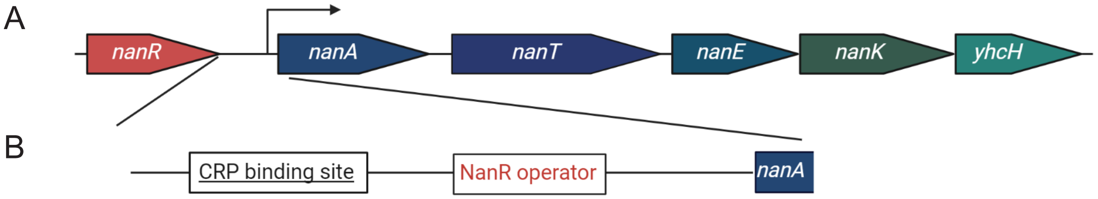

ACCCTGCTTTAGTGAACTCGATCGCAATATAAGCGTTCTGTCAGGATCATTGCGTAAACTGTCT
GGTATAACAGGTATAAAGGTATGCAGTCAGATAACACTGAATATGACAGAGGTCGTTATGGCAA
AAGATTTACGCGGCGTAATGCCGGCATTATTAACCCCATTCGATAACCAACAGCGGCTGGATGT
TGAAAGCTTGCGCCGTCTGGTGCGTTTCAA

Figure S6. The sialometabolic regulon in *C. rodentium* chromosome. (A) The *nan* operon consists of *nanATEK-yhcH* genes for sialic acid uptake and catabolism. The *nanR* gene encodes a repressor NanR that controls the expression of the *nan* operon. (B) Intergenic region between *nanR* and *nanA*. The nucleotide sequence between the end of *nanR* and the start codon of *nanA* (boxed) is indicated. The CRP binding site is underlined. The NanR operator containing GGTATA repeats is shown in red. Figure generated with Biorender.com.

Table S1. Virulence proteins secreted by *C. rodentium* in sialic acid-induced culture identified by LC-MS/MS.

Protein ID	Protein description	MW (kDa)	Unique peptides	Sequence coverage (%)
D2TV59	Putative serine protease autotransporter Pic	145.8	91	68.91
D2TV46	Putative serine protease autotransporter EspC	141.0	69	40.60
D2TKE2	T3SS translocator protein EspD	39.6	38	79.21
D2TKE8	Translocated intimin receptor Tir	56.3	34	82.82
D2TKE1	T3SS effector protein EspB	33.4	36	89.41
D2TKE3	T3SS translocator protein EspA	20.5	34	96.87
D2TKD7	T3SS effector protein EspF	30.9	22	71.10

Table S2. Primers for mutant construction.

Construct and primer designation	Primer sequence (5'-3') (restriction sites are underlined)
nanT deletion mutant	
nanT-P1	GTCTAGGTACCGCGTAAACTGTCTGGTATAACAGGTA (KpnI site)
nanT-P2	GTTGAGATGGCGATACCACGGGATGCTTTGGGTA
nanT-P3	GTGGTATCGCCATCTCAACGACGCTATTGACGGTAAGCCA
nanT-P4	GACAGTGAGCTCTGCAAATTGCCTTCCTGGATGAT (SacI site)
nanT-check-F	GTGCTGCACTATATGGATGTGGT
nanT-check-R	GATAATCGGTAGCGATACCAC
espC deletion mutant	
espC-P1	GACAGTGAGCTCATTCGGCTGGGTAAGGATTTGAGTGA (SacI site)
espC-P2	AATGCTGAGATTCATAACAATGTACGCTTT
espC-P3	GAATCTCAGCATTTTGTTCACAGCAAACCCTCCATG
espC-P4	GTCTAGGTACCATTATTTCAGCTGCTGTGCTAATACCT (KpnI site)
espC-check-F	CACAAGAAAAATGAAGCCCCT
espC-check-R	ACCTCAAAATCCCTGAAGACC
picC deletion mutant	
pic-P1	GTCTAGGTACCAGGATAACGGAGAACGGGATGAGCAG (KpnI site)
pic-P2	TTACGATAAGGACGGCATGAACGCGCAGATAAAGGACAACA
pic-P3	CCGTCCTTATCGTAAACCGGGATATCAGC
pic-P4	GACAGTGAGCTCAACAAATGGACTTAATAAGCAAC (SacI site)
pic-check-F	CTGAAAGGGTCTGTGTGAGTC
pic-check-R	ATGGAGGGTTTGCTGTGAATA

Table S3. Primers for qPCR analysis.

Target gene	Primer forward	Primer reverse
Il1b	CAGGATGAGGACATGAGCACC	CTCTGCAGACTCAAACTCCAC
Il6	GAGGATACCACTCCCAACAGACC	AAGTGCACTACTGTTGTTCATACA
Il17a	GCTCCAGAAGGCCCTCAGA	CTTTCCCTCCGCATTGACA
Ifng	TCAAGTGGCATAGATGTGGAAGAA	TGGCTCTGCAGGATTTTCATG
Rplp0	AGATTCGGGATATGCTGTTGGC	TCGGGTCCTAGACCAGTGTTC
